# Concordant nephrotic syndrome in twins with PAX2 and MYO1E mutations 

**DOI:** 10.5414/CNCS110799

**Published:** 2022-05-10

**Authors:** Oulimata K. Grossman, Claire F. Schretlen, Linda S. Nield

**Affiliations:** 1 Department of Pediatrics West Virginia University School of Medicine, Children’s Hospital Morgantown, WV, and; 2 Division of Nephrology, Nephrology Fellowship Program Johns Hopkins University School of Medicine Baltimore, MD, USA

**Keywords:** nephrotic syndrome, focal segmental glomerulosclerosis, paired box gene 2, PAX2-MYO1E

## Abstract

Introduction: The medical literature is scant with reports of twins diagnosed with nephrotic syndrome associated with genetic mutations. Mutations in the protein coding paired box gene 2 (*PAX2*) and in the non-muscle class I myosin, myosin 1E, (*MYO1E*) have been implicated in the development of steroid-resistant nephrotic syndrome. We describe the first case, to our knowledge, of the concordant presentation of nephrotic syndrome in twins with simultaneous mutations in *PAX2* and *MYO1E*. Case Report: At 32 months and 33 months of age, monochorionic, diamniotic twin girls presented with nephrotic syndrome. Each twin experienced three relapses during or after completion of corticosteroid treatment. Sustained remission was achieved with tacrolimus. Genetic testing of each twin revealed two heterozygous mutations of *MYO1E* and one homozygous mutation of *PAX2*. Renal biopsy results of one twin revealed pathologic findings consistent with minimal change nephropathy. The twins’ phenotypes have been essentially identical. Conclusion: Our cases add to the scant medical literature addressing nephrotic syndrome in twins with genetic mutations. Close monitoring of our unique patients will provide novel information about the clinical significance of combined mutations in *PAX2* and *MYO1E*.

## Introduction 

The term “nephrotic syndrome” (NS) emerged in 1883 and replaced the vague term “nephrosis” [1] to refer to the tetrad of heavy proteinuria (nephrotic range ≥ 40 mg/m^2^/h or urine protein/creatinine ratio ≥ 2,000 mg/g; or 3+ protein on urine dipstick), hypoalbuminemia (serum albumin level < 25 g/L), and edema associated with secondary elevated cholesterol. Nephrotic-range proteinuria is defined as a urine protein/creatinine ratio of > 2,000 mg/g (> 200 mg/mmol) in the absence of clinically overt NS [2, 3]. The disease process is the result of a defective glomerular filtration barrier basement which causes leakage of protein into the urinary space at a rate that is above the capacity of tubular reabsorption. One classification divides NS into primary (idiopathic) and secondary NS depending on the causal factor. Secondary causes are related to specific diseases of the glomerulus, tubules, and interstitial and vascular layers of the kidneys, as well as infections, toxins, and systemic diseases [4]. Among the genetic causes of NS is the paired box gene 2 *(PAX2)* mutation; myosin IE *(MYO1E)* mutation has also been implicated. The *PAX2* mutation has been implicated in the development of steroid-resistant NS in children, and the *MYO1E* mutation has been implicated in the development of a few cases of childhood focal segmental glomerulosclerosis (FSGS). 

Case reports of NS in twins have been relatively infrequently described, and reports of genetic mutations associated with twin cases of NS are even more scant in the medical literature. We describe the first case, to our knowledge, of the concordant presentation of NS in monochorionic, diamniotic twins at 32 – 33 months of age with simultaneous mutations in *PAX2* and *MYO1E*. 

## Case presentation 

Monochorionic, diamniotic twin girls born at 34 weeks and 5 days of gestation from an uncomplicated pregnancy presented ~ 6 weeks apart with NS. Table 1 provides the timeline of significant clinical events in each twin. Twin B was the first to present at 32 months of age after experiencing worsening facial and leg edema, abdominal discomfort, and decreased urine output, with a 2.3-kg weight gain over the previous 5 weeks. She had recently completed a course of oral antibiotics for otitis media; her parents reported that the presenting symptoms began following the onset of upper respiratory symptoms that accompanied the otitis media. Laboratory studies revealed severe proteinuria (15.657 mg/mg; normal ≤ 0.200 mg/mg), hypoalbuminemia (0.9 g/dL; normal 3.5 – 4.8 g/dL), and hypercholesterolemia (456 mg/dL; normal 125 – 228 mg/dL). Her creatinine level was normal at 0.31 mg/dL. Renal ultrasound was obtained, and findings were unremarkable. Further workup revealed a negative urine culture and negative titers for anti-streptolysin, anti-deoxyribonuclease-B and anti-nuclear antibodies. Screens for hepatitis A, B, and C were negative, and complement 3 and 4 levels were normal. Family history was negative for kidney disease and positive for common variable immunodeficiency in the mother and systemic lupus erythematosus in a distant cousin. Twin B was diagnosed with NS and treated with a prednisolone taper for a total of 6 weeks resulting in complete remission after 2 weeks. Three weeks after the first remission, twin B had her first relapse and was re-started on prednisolone, and again achieved remission in 2 weeks. She had another relapse and spontaneous remission during an upper respiratory tract infection, and while on a weaning-dose of steroids, she had a third relapse which was again treated with high-dose steroids. A renal biopsy was performed after completion of the steroid taper for this third relapse. The renal biopsy findings displayed minimal change nephropathy. Twin B was then started on a low dose of tacrolimus, ~ 9 months after her initial diagnosis. A genetic panel for NS, which tested for mutations in the genes *NPHS1*, *NPHS2*, *LAMB2*, *WT1*, *PLCE1*, *INF2*, *ACTN4*, and *TRPC6* was obtained. The results were positive for heterozygous mutations in two areas of the gene *MYO1E* (c.3146 C>A; p.P1049H heterozygous; c554 A>G, pD185G heterozygous) and one area of *PAX2* (c491C>A, p.T164N heterozygous); these mutations were interpreted as being of unknown significance. 

Twin A presented 41 days after her sister when she developed periorbital edema, and 3+ protein on a home urine dipstick. Like her sister, twin A had been treated with oral antibiotics for otitis media. Initial laboratory values were like those of twin B, revealing normal serum creatinine (0.39 mg/dL), hypoalbuminemia (2.2 g/dL), and hypercholesterolemia (234 mg/dL). Her renal ultrasound findings were normal. She was treated with prednisolone and went into remission after 10 days and received ~ 1 month of steroids; however, she experienced a relapse just 4 days after completion of the steroids. The patient had experienced 2 more relapses while on weaning courses of oral steroids. Due to her recurrent relapses, she was then started on tacrolimus concomitantly with her sister and was weaned off steroids after 8 weeks. Like her sister, she was relapse-free while taking tacrolimus. Both twins continued on tacrolimus until one twin developed a dystonic reaction after an increase in dosage following a low trough serum level of the drug. Tacrolimus was stopped in the affected twin, and rituximab successfully introduced after the treatment of another relapse of NS with high dose steroids, parents declined a trial of cyclosporine due to potential cosmetic side effects of this medication. The twins are currently on no medications and have been symptom free for 7 months. Genetic testing of twin A revealed the same mutations as those for twin B. The father also carried the same genetic mutations as the twins and had no renal or extrarenal manifestations. Further genetic work-up with whole genome sequencing was offered to the entire family, but the offer was declined. 

## Discussion 

Case reports of NS in identical twins have been described in the medical literature as far back as a half century ago [5]. In 2006, Ghiggeri et al. [6] described the discordant presentation and long-term outcomes of NS in two pairs of mono- and dizygotic twins who tested negative for the most common mutations associated with NS at that time. Our case report of NS in twins with an associated mutation in *PAX2* and *MYO1E* is a unique contribution to the medical literature. 

When the etiology of NS is of genetic origin, patients may also present with other manifestations that correlate with the discovered mutation(s). Despite having *PAX2* and *MYO1E* mutations, our twin patients presented with normal renal anatomy on ultrasound and no other extrarenal manifestations. Regarding *PAX2*, it has a significant role in nephrogenesis as well as in the development of other bodily organs and systems such as the eyes and the central nervous system. Mutation of this gene follows an autosomal dominant pattern. Early presentation of findings associated with the *PAX2* gene mutation has been described in infants and in children up to 14 years of age [7]. Most children diagnosed with this mutation also present with unilateral or bilateral renal hypoplasia, renal cysts, and extrarenal manifestations including eye complications [7]. FSGS without overt extrarenal manifestations has been noted in some adults and children with *PAX2* mutations [8, 9]. Iatropoulos et al. [10] described discordant phenotypes in monozygotic twins who had renal coloboma syndrome and a *PAX2* gene mutation and presented at different ages and had different progression rates. Regarding *MYO1E*, it encodes a non-muscle membrane-associated class I myosin which is reported to be a key component of the foot-process cytoskeleton and the podocyte structure in the glomerulus. It has also been reported to help glomerular capillaries to resist hydrostatic pressure at the glomerular filtration barrier through the generation of tension. Zhao et al. [11] reported steroid-resistant NS in a cohort of children with the *MYO1E* mutation in China. The *MYO1E* mutation has also been associated with FSGS in a group of pediatric patients with a homozygous mutation [12]. Autosomal recessive mutations have been linked to FSGS [13]. The role of the mutations in the occurrence of NS in our two patients is unknown at this time. 

The standard treatment of NS involves high-dose steroids: prednisone at an initial dose of 60 mg/24h/m^2^ (maximum dosage 80 mg/24h) in divided doses for 4 weeks, followed by a dose of 40 mg/24h/m^2^ in divided doses for three consecutive days out of 7 (for 4 weeks). In case of a relapse, the treatment followed the same prednisone regimen as described above, with the dose tapered after remission. Among children treated with steroids, some may respond well, some may be steroid-resistant, and some may be non-responders who, despite 5 weeks of steroid treatment, will not go into remission. Among patients who respond with total remission for 3 consecutive days, a few will manifest frequent relapses. Steroid-dependent children are expected to have 2 consecutive relapses during steroid therapy or within 14 days of finishing therapy. For children with NS who experience frequent relapses, the Kidney Disease Improving Global Outcomes Guidelines recommend a course of calcineurin inhibitors (cyclosporin or tacrolimus) for 12 months while monitoring for possible medication toxicity [14]. Cosmetic side effects, such as hypertrichosis, are possible with cyclosporin. Rituximab, a chimeric anti-CD20 monoclonal antibody and its biosimilars, is an additional option for the treatment of children with idiopathic NS who fail to respond to calcineurin inhibitors [15]. Our twins were treated initially with the standard protocol for NS but developed a pattern of steroid dependency and frequent relapse. The medication switch to a calcineurin inhibitor, specifically tacrolimus, was halted by the side effects experienced by one of the twins. 

## Conclusion 

The concomitant presence of *PAX2* and *MYO1E* mutations in a set of twins with NS has not been previously described. Our patients developed steroid dependence and responded well to tacrolimus and to rituximab. Despite the presence of genetic mutations that could predispose them to FSGS, they are still in remission. Close monitoring of our two patients will provide insight into the clinical significance and long-term outcomes of the finding of these two mutations occurring simultaneously in the same individual. 

## Acknowledgment 

The authors acknowledge Sherry Kanosky, Tammy Glover, and Dr. Gaurav Nanda for their contributions to the care of the patients and their contributions to information included in the manuscript. 

## Funding 

The authors have received no funding concerning this publication. 

## Conflict of interest 

The authors certify that they have no conflict of interest concerning this article. 

**Table 1.** Timeline of significant clinical events in each twin.

	Twin A	Twin B
Date of initial presentation Age at initial presentation; symptoms and signs Initial albumin level (normal 3.4 – 4.8 g/dL) Initial urine analysis microscopy Random UPC (normal ≤ 0.200 mg/mg) Initial cholesterol level (normal 125 – 228 mg/dL) Initial dose of steroids	**January 21, 2019 ** 32 months and 23 days; periorbital edema 2.2 g/dL 3 RBCs/HPF and 5 WBCs/HPF 14 mg/mg 234 mg/dL 2 mg/kg/day or 60 mg/m^2^/day	**December 7, 2018 ** 31 months and 8 days; facial and leg edema, abdominal discomfort 0.9 g/dL > 182 RBCs/HPF and 6 WBCs/HPF 15.6 mg/mg 456 mg/dL 2 mg/kg/day or 60 mg/m^2^/day
Date of first remission	**February 1, 2019**	**December 24, 2018**
Date of first relapse	**April 12, 2019**	**April 16, 2019**
Steroid dose for subsequent relapse treatments	2 mg/kg/day or 60 mg/m^2^/day	2 mg/kg/day or 60 mg/m^2^/day
Date of second remission	**April 18, 2019**	**April 29, 2019**
Date of second relapse	**May 2, 2019**	**May 16, 2019**
Date of third remission	**May 9, 2019**	**May 21, 2019**
Date of third relapse	**July 5, 2019**	**July 24, 2019**
Fourth remission	**July 15, 2019**	**August 15, 2019**
Biopsy findings	N/A	MCD
Date of initial tacrolimus dose Tacrolimus trough level	**August 20, 2019 ** 6.6 ng/mL	**August 20, 2019 ** 7.4 ng/mL
Date of final relapse	**October 15, 2020**	N/A
Date of dystonic reaction	N/A	November 26, 2020*
Number of rituximab doses (3.75 mg/m^2^/dose)	3 weekly doses plus 4^th^ dose 6 months later with re-population of CD19	3 weekly doses plus 4^th^ dose 6 months later with re-population of CD19

*Tacrolimus trough level was 5.5 ng/mL; target level was 5 – 7 ng/mL. RBCs = red blood cells; HPF = high-power field; WBCs = white blood cells; UPC = urine protein to creatinine ratio; MCD = minimal change disease.
